# Supplementary material for: Treatment Reducing Endothelial Activation Protects against Experimental Cerebral Malaria
Source: Pathogens. 2022 Jun 2;11(6):643. doi: 10.3390/pathogens11060643 (PMC9229727; doi:10.3390/pathogens11060643)
Supplement: Supplementary file 1 [file pathogens-11-00643-s001.zip › pathogens-1737443-supplementary.pdf]

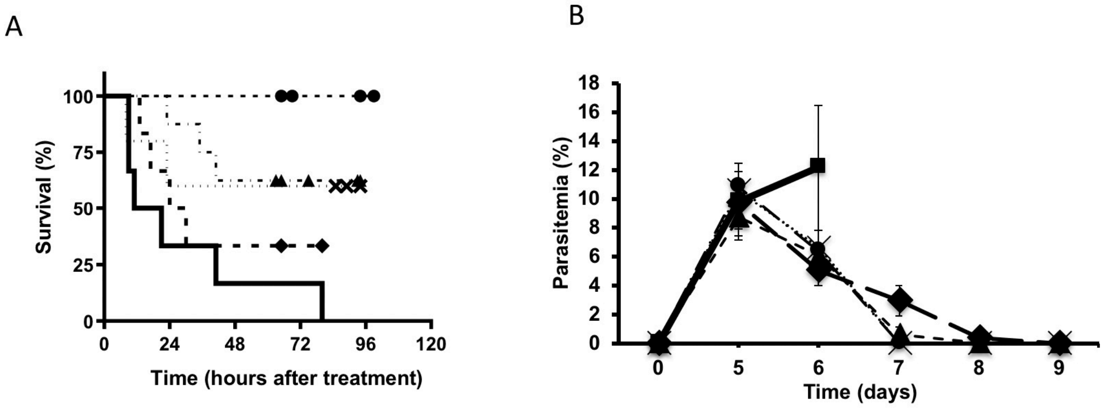

Figure S1. Artesunate dose selection. Artesunate dose of 5 mg/Kg/day was selected after running a prospective study in C57BL/6J mice infected with *P. berghei* ANKA and treated after developing signs of CM (score ≥ 3) with different doses of artesunate: 40 mg/Kg/day (circle), 20 mg/Kg/day (cross), 10 mg/Kg/day (triangle), 5 mg/Kg/day (diamond) and 1 mg/Kg/day (square). Only those mice treated at the lowest doses of 5 and 1 mg/Kg/day showed a survival rate below 40% (**A**) but the lowest dose of 1 mg/Kg/day were not effective in clearing the parasitemia (**B**).

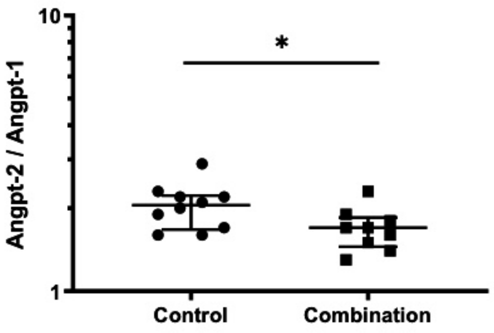

Figure S2. Angpt-2/Angpt-1 ratio. End-point plasma levels Angpt-2/Angpt-1 ratio, expressed as log base 10, in CB57/B6 mice infected with *P. berghei* ANKA and treated with vehicle (Control) or with irbesartan 50 mg/Kg/day and atorvastatin 10 mg/Kg/day (Combination) from day one of infection. Angiopoietin-2 (Angpt-2) Angiopoietin-1 (Angpt-1). Student's t-test * $p = 0.0388$ vs. control.

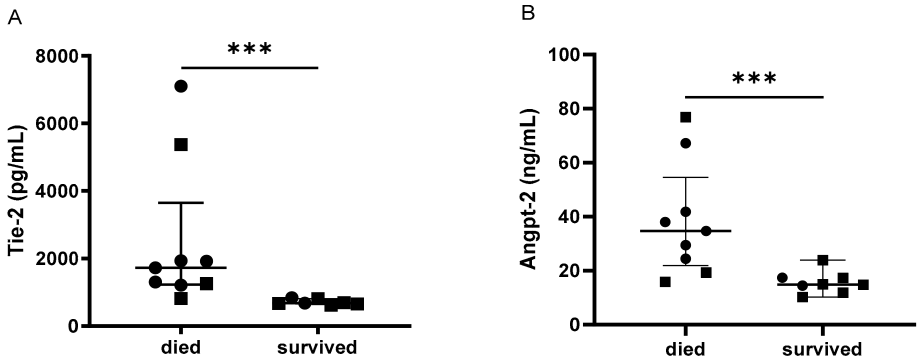

Figure S3. Comparison of levels of markers of endothelial cell activation between mice that died and survived. End-point plasma levels of Tie-2 (**A**) and Angiopoietin-2 (**B**) in C57BL/6J mice infected with *P. berghei* ANKA and treated after developing signs of CM (score ≥ 3) with artesunate 5mg/Kg/day alone (circles, n = 8) or artesunate plus the atorvastatin/irbesartan combination treatment (squares, n = 9). Wilcoxon signed-rank test, *** $p = 0.0003$ and *** $p = 0.0010$ for **A** and **B**, respectively.
